# Association between health anxiety dimensions and preventive behaviors during the COVID-19 pandemic among Japanese healthcare workers

**DOI:** 10.1016/j.heliyon.2023.e22176

**Published:** 2023-11-10

**Authors:** Kentaro Nagao, Takuya Yoshiike, Ryo Okubo, Kentaro Matsui, Aoi Kawamura, Muneto Izuhara, Tomohiro Utsumi, Megumi Hazumi, Mio Shinozaki, Ayumi Tsuru, Yohei Sasaki, Kazuyoshi Takeda, Hirofumi Komaki, Hideki Oi, Yoshiharu Kim, Kenichi Kuriyama, Hidehiko Takahashi, Takeshi Miyama, Kazuyuki Nakagome

**Affiliations:** a Department of Sleep-Wake Disorders, National Institute of Mental Health, National Center of Neurology and Psychiatry, 4-1-1 Ogawa-Higashi, Kodaira, Tokyo, 187-8553, Japan; b Department of Psychiatry, National Center Hospital, National Center of Neurology and Psychiatry, 4-1-1 Ogawa-Higashi, Kodaira, Tokyo, 187-8553, Japan; c Department of Psychiatry and Behavioral Sciences, Graduate School of Medical and Dental Sciences, Tokyo Medical and Dental University, 1-5-45 Yushima, Bunkyo-ku, Tokyo, 113-8510, Japan; d Clinical Research & Education Promotion Division, National Center Hospital, National Center of Neurology and Psychiatry, 4-1-1 Ogawa-Higashi, Kodaira, Tokyo, 187-8553, Japan; e Department of Laboratory Medicine, National Center Hospital, National Center of Neurology and Psychiatry, 4-1-1 Ogawa-Higashi, Kodaira, Tokyo, 187-8553, Japan; f Department of Behavioral Medicine, National Institute of Mental Health, National Center of Neurology and Psychiatry, 4-1-1 Ogawa-Higashi, Kodaira, Tokyo, 187-8553, Japan; g Department of Surgery, National Center Hospital, National Center of Neurology and Psychiatry, 4-1-1 Ogawa-Higashi, Kodaira, Tokyo, 187-8553, Japan

**Keywords:** Awfulness, COVID-19, Health anxiety, Preventive behavior, Self-protection

## Abstract

**Objective:**

Health anxiety (HA), defined as excessive worry about having a serious medical condition, may affect preventive behaviors during the coronavirus disease 2019 (COVID-19) pandemic. We examined the distinct role of two dimensions of HA—perceived likelihood (probability dimension) and awfulness of illness (awfulness dimension)—in self-protection, as reflected in preventive behaviors during the pandemic.

**Methods:**

Participants comprised 657 healthcare workers. Data were collected between February 24 and 26, 2021. The Short Health Anxiety Inventory determined the HA dimensions. Adherence to the government's recommendations for COVID-19 preventive behaviors was self-rated. An independent association between each HA dimension and participants' adherence to the recommendations was examined using multivariable regression.

**Results:**

Within the analyzed sample of 560 subjects, severe HA was observed in 9.1 %. The more the participants felt awful, the less frequently they engaged in the recommended preventive behaviors (adjusted odds ratio = 0.993, 95 % confidence interval: 0.989, 0.998, p = 0.003) regardless of their profession, working position, psychological distress, sleep disturbance, and current physical diseases. However, the probability dimension was not associated with their preventive behaviors.

**Conclusion:**

The awfulness dimension of HA could be a more sensitive marker of preventive behaviors than the probability dimension. Paying particular attention to the awfulness dimension may help optimize self-protection strategies during the COVID-19 pandemic. A two-dimensional understanding of HA may be useful for the maintenance of the healthcare system and public health as well as healthcare workers’ own health.

## Highlights

• The role of two health anxiety dimensions in COVID-19 prevention was examined.
• Only the awfulness dimension was found to be associated with preventive behaviors.
• The more participants felt awful, the less they followed recommended behaviors.
• The awfulness dimension may be more important for self-protection during the pandemic.

## 1 Introduction

Health anxiety (HA) is defined as the obsessive and irrational worry about one's health, with the individual investing a significant amount of time in seeking help and medical care. This is driven by a sense of worry over getting ill or illness, even when there are no apparent symptoms or only minor symptoms [1,2]. Although many of us have experienced HA in our lives during difficult circumstances, it may become pathological when it exceeds a normal level and impedes normal functioning in daily life. Despite an ongoing debate [3], existing theories posit two major dimensions of HA: one is related to “the perception of the likelihood of an illness (probability dimension),” whereas the other is more closely associated with “the anticipation of the burden of being ill (awfulness dimension)” [4,5]. HA may thus depend on the individual's risk of falling ill, through home and workplace hygiene, dietary habits, current or past history of illnesses, and family history of serious hereditary conditions. These risk factors make estimating the probability and awfulness of a severe disease complex. One way to overcome this difficulty would be to understand the association between HA and self-protection behavior by investigating the behavioral response to a disease that can place most individuals at risk. The coronavirus disease 2019 (COVID-19) pandemic provides a chance to estimate the influence of HA on behavioral responses as everyone was equally on alert with regard to the possibility of infection.

Since the COVID-19 pandemic started in March 2020, the disease has instilled fear in people worldwide, as it causes severe respiratory damage [6]. In Japan, there were 2,225,763 cases of infection and 18,540 deaths up to January 25, 2022, with a fatality rate of 0.8 %, comparable to the global average. Until the first half of 2021, since no specific treatment had been established and vaccines provided limited protection, virtually all individuals were seriously afraid of COVID-19 infection. In particular, healthcare workers (HCWs) felt a more emergent crisis owing to their frequent contact with patients experiencing respiratory infection, including COVID-19 [7]. Consequently, they have a higher likelihood of experiencing fear and anxiety around COVID-19 than people engaged in other occupations [8]. However, their fear is expected to be relatively rational because they have in-depth knowledge about how to protect themselves from COVID-19 infection.

HA may involve behavioral responses to the COVID-19 pandemic. It might lead to infection prevention behaviors, such as hand hygiene and social distancing, or it may help individuals detect the early signs and symptoms of COVID-19, thereby increasing the likelihood of survival. However, it is also likely that persistent and/or greater HA compared with the objective severity of the threat may paradoxically lead to maladaptive strategies. Safety-seeking behaviors are directly driven by the inadequate anticipation of a burden of being ill, caused by catastrophic misinterpretations of bodily sensations or dysfunctional beliefs about health and illness. However, previous studies have reported inconsistent results regarding whether HA relates to adaptive or maladaptive preventive behaviors [[9], [10], [11], [12], [13]]. This inconsistency may have been affected by the fact that most previous studies in nonclinical samples considered HA a behavior that reflects a single severity continuum that is not considered from the awfulness dimension, a core cognition of HA [4]. Therefore, it remains unclear which dimension of HA is more closely related to maladaptive self-protection strategies. Given that the awfulness dimension could be more directly associated with negative emotions (e.g., aversion) [4], it may be more prone to involvement in maladaptive self-protection strategies than the probability dimension.

The optimization of self-protection strategies is of particular importance not only for one's own health, but also for the maintenance of the healthcare system and public health. In particular, HCWs are required to strictly follow the self-protection protocol for maintaining their own health and public health systems. Against this background, we aimed to examine the individual roles of the two dimensions of HA in self-protection behavior among HCWs during the COVID-19 pandemic in Japan.

## 2 Methods

### 2.1 Design and participants

This cross-sectional survey was conducted from February 24 to 26, 2021. A total of 657 participants were recruited from all staff working at the National Center of Neurology and Psychiatry who received the research announcements at the workplace and volunteered to participate in the study. The inclusion criteria were as follows: (1) individuals employed by the National Center of Neurology and Psychiatry and (2) individuals who could understand the explanation and provide written informed consent. The exclusion criterion was (1) individuals with any symptoms over the past 10 days, such as fever (37.5 °C or higher), respiratory symptoms, severe malaise, and change in sense of smell or taste. A total of 657 people participated in the study. Of these, 97 participants had missing data for their questionnaires, which resulted in 560 valid observations. For a multivariable regression with 14 predictors, a power analysis conducted in G*Power yielded a sample size of 557 with the following settings: power at 95 %, alpha level at 0.05, and a small effect size (f = ·05); thus, our study demonstrated adequate power.

### 2.2 Ethical considerations

The study protocol was reviewed and approved by the Ethics Committee of the National Center of Neurology and Psychiatry (approval number A2020-121). This study was performed in accordance with the Declaration of Helsinki. Prior written informed consent was obtained from all participants.

### 2.3 Procedure

Participants completed a self-administered questionnaire before blood collection for COVID-19 antibody testing. The questionnaire consisted of sociodemographic, psychological, and COVID-19-related information, as well as adherence to COVID-19 preventive behaviors. The coronavirus infection was confirmed by the Elecsys Anti-SARS-CoV-2 RUO (Roche Diagnostics K.K., Inc, Tokyo), which uses antibodies to determine whether an individual has had a past infection with the severe acute respiratory syndrome coronavirus 2, with a sensitivity and specificity of 99.5 % and 99.8 %, respectively.

### 2.4 Measures

#### 2.4.1 Health anxiety

The Short Health Anxiety Inventory (SHAI) is a widely used 18-item self-rated measure of HA over the past six months, with prior evidence of good internal consistency (Cronbach's alpha ranges from 0.74 to 0.96 [3]), that allowed us to separately assess the two distinct dimensions of HA. We used a validated Japanese version of the SHAI [14]. The SHAI consists of two subscales assessing 1) the probability dimension: the perceived likelihood of becoming seriously ill (SHAI-IL, 14 items) and 2) the awfulness dimension: the perceived negative consequences of being seriously ill (SHAI-NC, four items) [5,14]. Each item consists of four possible statements rated from 0 to 3 (0 = “*I do not worry about my health*,” 3 = “*I spend most of my time worrying about my health*”). Item scores are summed to obtain the scores of these two subscales, with higher scores indicating higher levels of the respective dimensions of HA. The total scores of the SHAI-IL and SHAI-NC subscales range from 0 to 42 and 0 to 12, respectively. An average total score (18 items) of 14.04 ± 6.90 [mean ± standard deviation (SD)] was found in nonclinical samples for the Japanese version [14]. In this study, Cronbach's alpha values were 0.86 for the total scale, 0.86 for the SHAI-IL, and 0.75 for the SHAI-NC.

#### 2.4.2 Adherence to recommendations for COVID-19 preventive behaviors

Individual levels of adherence to the Japanese government's recommendations for COVID-19 preventive behaviors were assessed using a four-item self-report measure specifically created for this study. In Japan, after the first confirmed case of COVID-19 on January 16, 2020, the first national state of emergency was declared on April 16. Since then, the Japanese government has recommended adhering to the following four infection prevention behaviors: 1) specifically avoiding three places/situations (*three Cs*: closed spaces with poor ventilation, crowded places with many people nearby, and close-contact settings such as close-range conversations); 2) staying at least 2 m away from others; 3) wearing a mask when talking outside or indoors; and 4) adhering to hand hygiene (hand washing and hand disinfecting). In this study, participants were asked to rate their levels of frequency of the four preventive behaviors on a four-point scale, with responses ranging from 1 (never) to 4 (always). The total score, a sum of each item score, ranged from 4 to 16, and was used to index the levels of general adherence to the recommendations, as reported previously [[15], [16], [17]], with higher scores indicating higher levels of adherence. Internal consistency was acceptable in this sample (α = 0.62).

#### 2.4.3 COVID-19-related information

Participants' vaccination status was self-reported. Participants who had been involved in treating patients with COVID-19 were defined as frontline workers. History of close contact with a COVID-19 case was defined as spending more than 15 min within 1 m of a person with COVID-19; direct physical contact with a person with COVID-19; providing direct care for patients with COVID-19 or being in direct contact with the patient's secretions without using proper personal protective equipment; or living with someone who had COVID-19.

#### 2.4.4 Other covariates

Sociodemographic covariates included participants' age, gender, body mass index, exercise habits (<1 h per week, ≥1 h per week), smoking status (never, former, current), and alcohol intake (non-drinkers: <1 day/month, occasional drinkers: 1–3 days/month, regular drinkers: 1–2 days/week or more) [18]. Current physical disease was considered positive if participants reported having at least one physical condition that required medical assessments or treatments. Additionally, participants’ professions in the area of healthcare were reported and categorized into six groups: director; doctor; nurse; other medical staff, including physical and occupational therapists, psychologists, pharmacists, and lab technicians; medical assistants; and other professions, such as those related to research, education, cleaning, or food service.

Psychological distress was measured by the Japanese version of the Kessler Psychological Distress Scale [19]. We considered participants with a total score (0‒24) ≥ 5 as having significant psychological distress [20]. Perceived sleep disturbance was measured with the Japanese version of the Pittsburgh Sleep Quality Index [21,22]. We considered participants with a total score (0‒21) ≥ 6 as having subjective sleep disturbance [23].

### 2.5 Statistical analysis

In the sample (N = 657), 14.7 % (n = 97) had at least one missing value for any of the predictor or outcome variables or covariates derived from the aforementioned questionnaire. Therefore, we conducted a complete case analysis because it could provide less biased results than multiple imputation in this case [24]. We intended to determine whether and how the two HA dimensions were associated with COVID-19 preventive behaviors. Confirmatory factor analysis, with each factor considered orthogonal, was first performed to confirm whether a two-factor model of HA would be supported in this sample, as previously confirmed in the original [5] and Japanese student samples [14]. The global fit was evaluated using the comparative fit index (CFI), standardized root mean square residual (SRMR), and root mean square error of approximation (RMSEA). Values of CFI ≥0.90, SRMR ≤0.08, and 0.03 ≤ RMSEA ≤0.08 were considered adequate [25,26].

As the distribution of behavioral adherence scores was skewed (skewness = −0.73; kurtosis = 0.89), the relationship between HA dimensions and preventive behaviors was analyzed in the context of the generalized linear model (GLM) with gamma distribution and log link function. Possible adverse or protective effects of these two HA dimensions on adherence to the recommendations for preventive behaviors were tested in a univariable GLM, with the adherence score as a single dependent variable, and SHAI-IL and SHAI-NC scores and other covariates as predictors. Variance inflation factor values obtained to identify multicollinearity between predictors in the adjusted model fell between 1.06 and 1.47, which is generally considered acceptable. Next, independent associations of the two HA dimensions with preventive behaviors were tested in a multivariable GLM adjusted for mental health status, COVID-19-related covariates, and sociodemographic covariates. Given the extremely low rates of participants with positive COVID-19 antibodies and those who had been vaccinated, these variables were not considered as covariates in our regression analyses. The results were presented as odds ratios (ORs), which are the exponentiated coefficients of GLM, with 95 % confidence intervals (CIs). Given that whether participants already had an illness could affect the relationship between HA dimensions and preventive behaviors, we ran the same GLM as a sensitivity analysis, while excluding participants with current physical diseases (n = 144, 25.7 %). We replaced the missing data using multiple imputation by chained equations with 20 imputed datasets and thereafter compared the results with those from the complete case analysis [27].

All analyses were performed using SPSS version 23 (IBM Corp., Armonk, NY, USA) and Amos version 26 (SPSS Inc., Chicago, IL, USA). A p-value <0.05 was considered statistically significant.

## 3 Results

### 3.1 Participants’ characteristics

Table 1 presents the participants’ sociodemographic, COVID-19-related, and psychological characteristics. Most participants had neither been vaccinated for COVID-19 (n = 559, 99.8 %) nor infected by severe acute respiratory syndrome coronavirus 2 (n = 556, 99.3 %). A quarter of the participants were considered frontline workers (n = 144, 25.7 %). A small proportion of participants had had close contact with people with COVID-19 (n = 20, 3.6 %). A considerable proportion of participants had moderate (n = 192, 34.3 %) to severe (n = 43, 7.7 %) psychological distress and sleep disturbance (n = 211, 37.7 %).

**Table 1:** Participants’ characteristics.

Characteristic	Complete case sample (N = 560)
Age, mean (SD)	42.33 (10.72)
Gender, n (%)	
Men	186 (33.21)
Women	374 (66.79)
Profession, n (%)	
Director	4 (0.71)
Doctor	36 (6.43)
Nurse	166 (29.64)
Other medical staff	114 (20.36)
Medical assistant	16 (2.86)
Others	224 (40.00)
Working position	
Frontline	144 (25.71)
Second-line	416 (74.29)
History of close contact with COVID-19 cases, n (%)	
Yes	20 (3.57)
No	540 (96.43)
COVID-19 vaccination status, n (%)	
Vaccinated	1 (0.18)
Unvaccinated	559 (99.82)
COVID-19 antibodies, n (%)	
Positive	4 (0.71)
Negative	556 (99.29)
Body mass index, mean (SD)	22.20 (3.53)
Smoking status, n (%)	
Never	413 (73.75)
Former	99 (17.68)
Current	48 (8.57)
Alcohol intake, n (%)	
Non-drinkers (<1 day/month)	213 (38.04)
Occasional drinkers (1–3 days/month)	106 (18.93)
Regular drinkers (1–2 days/week or more)	241 (43.04)
Current physical disease, n (%)	
Yes	144 (25.71)
No	416 (74.29)
Exercise habits, n (%)	
<1 h per week	366 (65.36)
≥1 h per week	194 (34.64)
Psychological distress, K6 score, mean (SD)	4.67 (4.68)
Sleep disturbance, PSQI score, mean (SD)	5.11 (2.88)
SHAI total score, mean (SD)	14.55 (6.04)
Illness likelihood subscale score, mean (SD)	10.93 (5.15)
Negative consequence subscale score, mean (SD)	3.62 (1.96)

K6, Kessler Psychological Distress Scale; PSQI, Pittsburgh Sleep Quality Index; SD, standard deviation; SHAI, Short Health Anxiety Inventory; COVID-19, coronavirus disease 2019

The levels of HA dimensions were comparable to those reported in the Japanese student sample, although the awfulness dimension of HA was slightly increased. The SHAI-IL and SHAI-NC scores [mean (SD)] were 10.9 (5.1) and 3.6 (2.0), respectively, and the SHAI total score was 14.6 (6.0) (Table 1). With a total cutoff score of 23, which is reported to accurately distinguish nonclinical community individuals from patients with severe HA or hypochondriasis [28], 51 (9.1 %) participants were considered to have severe HA.

Participants reported that during the pandemic, they had generally adhered to the Japanese government's recommendations on COVID-19 preventive behaviors, as indicated by a mean (SD) total score of 14.3 (1.3) (Table 2).

**Table 2:** Levels of adherence to recommendations for preventive behaviors.

		Number of responses (%)			
	Mean (SD)	Never	Seldom	Usually	Always
Avoiding three Cs, score	3.5 (0.5)	0 (0)	12 (2.1)	263 (47.0)	285 (50.9)
Social distancing, score	3.2 (0.6)	2 (0.4)	57 (10.2)	356 (63.6)	145 (25.9)
Mask hygiene, score	3.8 (0.4)	0 (0)	3 (0.5)	82 (14.6)	475 (84.8)
Hand hygiene, score	3.8 (0.4)	0 (0)	5 (0.9)	87 (15.5)	468 (83.6)
General adherence, total score	14.3 (1.3)	–	–	–	–

SD, standard deviation.

### 3.2 Association of HA dimensions with preventive behaviors

Confirmatory factor analysis of SHAI item scores showed that the two-factor model of HA had an adequate fit (CFI = 0.90, SRMR = 0.050, RMSEA = 0.061 [90 % CI: 0.054, 0.067]). Factor loadings for each SHAI item are reported in Table 3.

**Table 3:** Factor loadings for the Short Health Anxiety Inventory item scores.

	Loading	
	Factor 1:	Factor 2:
Short Health Anxiety Inventory item	Probability of illness	Awfulness of illness
Illness likelihood subscale		
1. Time spent worrying about health	.536	.185
2. Noticing aches and pains	.230	.139
3. Awareness of bodily sensations/changes	.435	.153
4. Ability to resist thoughts of illness	.560	.366
5. Fear of having serious illness	.745	.230
6. Picturing self being ill	.657	.272
7. Ability to take mind off health thoughts	.562	.179
8. Relieved if doctor says that nothing is wrong	.496	.313
9. Hear about illness and think I have it	.630	.267
10. Wonder what body sensations/changes mean	.389	.232
11. Feeling at risk for developing illness	.620	.235
12. Think I have serious illness	.798	.255
13. Ability to think of other things if I notice unexplained body sensation	.511	.342
14. Family/friends say I worry about my health	.112	.015
Negative consequence subscale		
15. Ability to enjoy life if I have an illness	.222	.694
16. Chance of medical cure if I have an illness	.249	.579
17. Illness would ruin aspects of life	.201	.766
18. Loss of dignity if I had an illness	.225	.592

The two HA dimensions were associated differently with infection prevention behaviors: the more intensely participants anticipated negative consequences of being ill, the less frequently they adhered to the recommendations for prevention behaviors; however, no statistically significant association was found between the perceived illness likelihood and preventive behaviors. SHAI-NC scores were negatively associated with adherence scores of preventive behaviors (adjusted OR = 0.993, 95 % CI: 0.989, 0.998, p = 0.003). However, SHAI-IL scores were not significantly associated with adherence scores of preventive behaviors (adjusted OR = 1.001, 95 % CI: 0.999, 1.003, p = 0.18). The observed association was independent of the effect of history of close contact with individuals who had contracted COVID-19 (adjusted OR = 1.072, 95 % CI: 1.027, 1.118, p = 0.001) and occupation (nurse: adjusted OR = 0.966, 95 % CI: 0.946, 0.987, p = 0.001) on preventive behaviors. Neither psychological distress (adjusted OR = 1.000, 95 % CI: 0.998, 1.002, p = 0.802) nor subjective sleep disturbance (adjusted OR = 1.000, 95 % CI: 0.987, 1.004, p = 0.800) was significantly associated with preventive behaviors (Table 4). The sensitivity analysis found that these associations between HA dimensions and preventive behaviors remained constant among participants without current physical diseases (Table S1). The results from the data supplemented via multiple imputation for missing values did not differ substantially from the complete case analysis (Table S2).

**Table 4:** Factors associated with preventive behaviors among participants (N = 560).

	Crude model		Adjusted model	
Variable	OR (95 % CI)	*p*	OR (95 % CI)	*p*
Age	1.001 (1.000, 1.001)	0.084	1.000 (0.999, 1.001)	0.830
Gender				
Men	1 (ref)		1 (ref)	
Women	0.996 (0.979, 1.012)	0.606	0.997 (0.979, 1.015)	0.709
Health anxiety				
Illness likelihood subscale	1.000 (0.999, 1.002)	0.534	1.001 (0.999, 1.003)	0.182
Negative consequence subscale	0.994 (0.990, 0.998)	**0.003**	0.993 (0.989, 0.998)	**0.003**
Psychological distress	1.000 (0.998, 1.001)	0.761	1.000 (0.998, 1.002)	0.802
Sleep disturbance	1.000 (0.997, 1.003)	0.901	1.000 (0.987, 1.004)	0.800
Profession				
Director	1.087 (0.990, 1.192)	0.080	1.086 (0.991, 1.191)	0.078
Doctor	1.019 (0.986, 1.054)	0.253	1.014 (0.978, 1.052)	0.437
Nurse	0.968 (0·950, 0·986)	**0·001**	0·966 (0.946, 0.987)	**0·001**
Other medical staff	0.978 (0.957, 0.999)	**0.039**	0.979 (0.958, 1.001)	0.056
Medical assistant	0.970 (0.925, 1.017)	0.212	0.976 (0.931, 1.023)	0.311
Others	1 (ref)		1 (ref)	
Working position				
Frontline	1.004 (0.986, 1.023)	0.633	1.005 (0.986, 1.025)	0.601
Second-line	1 (ref)		1 (ref)	
History of close contact with COVID-19 cases				
Yes	1.047 (1.003, 1.092)	**0.036**	1.072 (1.027, 1.118)	**0.001**
No	1 (ref)		1 (ref)	
Body mass index	1.001 (0.999, 1.003)	0.405	1.000 (0.998, 1.002)	0.958
Smoking status				
Never	1 (ref)		1 (ref)	
Former	1.014 (0.993, 1.036)	0.188	1.017 (0.996, 1.039)	0.120
Current	0.974 (0.947, 1.003)	0.075	0.980 (0.951, 1.009)	0.180
Alcohol intake				
Non-drinkers (<1 day/month)	1 (ref)		1 (ref)	
Occasional drinkers (1–3 days/month)	0.999 (0.977, 1.021)	0.915	0.998 (0.976, 1.020)	0.830
Regular drinkers (1–2 days/week or more)	0.999 (0.981, 1.016)	0.874	0.998 (0.980, 1.015)	0.792
Current physical diseases				
Yes	0.016 (0.997, 1.034)	0.094	1.008 (0.989, 1.027)	0.400
No	1 (ref)		1 (ref)	
Exercise habits				
<1 h per week	1 (ref)		1 (ref)	
≥1 h per week	1.012 (0.996, 1.029)	0.146	1.006 (0.989,1.022)	0.503

OR, odds ratio; CI, confidence interval; COVID-19, coronavirus disease 2019.

## 4 Discussion

We aimed to understand the role of the two dimensions of HA in adherence to the recommendations for COVID-19 preventive behaviors among HCWs one year after the start of the pandemic. As the Japanese government had neither begun COVID-19 vaccinations, nor offered antiviral medications for COVID-19, strict adherence to recommended preventive measures was likely to be the exclusive key to preventing COVID-19. In accordance with previous findings, a two-factor model of HA consisting of perceived likelihood (probability dimension) and awfulness of illness (awfulness dimension) was supported in our sample. We found that the awfulness dimension of HA (SHAI-NC) indicated a risk factor for decreased adherence to the recommendations for preventive behaviors, whereas the probability dimension of HA (SHAI-IL) served as neither a risk, nor protective factor for preventive behaviors.

To the best of our knowledge, this study is the first to find an independent negative association between the awfulness dimension of HA and COVID-19 preventive behaviors: the more a person anticipates the burden of being ill, the less frequently the person engages in the recommended preventive behaviors. This finding indicates that HCWs who overestimate the likelihood of illness may not necessarily adhere to the behavioral recommendations. In contrast, previous studies have provided evidence for the positive association between HA and safety-seeking behaviors using nonclinical community samples [9,10,12,13], although there is evidence for the negative association between the two [13]. These findings partly support the notion that individuals who experience a greater threat are more likely to engage in corresponding safety-seeking behaviors. This positive association could be mainly attributed to the association of the probability dimension, rather than the awfulness dimension of HA, with safety-seeking behaviors. These studies only examined the probability dimension or SHAI total score for 18 items, and the awfulness dimension was not separately considered. However, we did not detect either a significant facilitative or an impeding effect of the probability dimension on preventive behaviors. One possible explanation is that there was less variation among individuals because the items included in the probability dimension seem to represent a rational concern under the circumstances of the COVID-19 pandemic. In contrast, a person will likely engage poorly in preventive behaviors, typically when they feel excessive awfulness about the negative consequences of an illness (e.g., COVID-19) raising the sense of uncertainty. Moreover, our results include implications for more appropriate methods of assessing HA. In nonclinical samples, where adaptive aspects of HA would be relatively well preserved, the maladaptive aspects of HA may be poorly extracted, especially when using the probability dimension. However, our results suggest that the awfulness dimension may enable a more sensitive assessment of maladaptive coping.

The findings revealed that participants with a history of close contact with COVID-19 adhere to preventive behaviors than those without, which aligns with a previous study [29]. Meanwhile, we observed that nurses tended to have lower adherence to preventive behaviors than the reference group. This finding contradicts previous studies showing a protective role nurses have in preventive behaviors during the pandemic [30]. Although the reason for the discrepancy is unknown, given that nurses have been reported to be among the most distressed or threatened occupations in the pandemic [31], it is plausible that they have more maladaptive behavioral outcomes. Furthermore, even HCWs with appropriate knowledge of COVID-19 threats and related preventive behaviors have been suggested to demonstrate an elevated awfulness dimension of HA and subsequent maladaptive behavior. However, because the nature of COVID-19 was still unclear during the period when the survey was conducted, most people in Japan remained extremely cautious of COVID-19, and HCWs were vigilant about the risk of getting ill. This may have enhanced the awfulness dimension of HA. Therefore, careful public statements that do not overstate the warning when controlling an infectious disease pandemic, such as COVID-19, are warranted. Many participants had moderate or higher levels of psychological distress and significant sleep problems. To be sure, burnout is a problem with the COVID-19 pandemic [[32], [33], [34]]. Although we observed that the awfulness dimension of HA was associated with preventive behaviors independently from psychological distress and subjective sleep disturbance—both of which are closely related to burnout—we cannot rule out the possibility that decreased compliance with preventive behaviors resulted from burnout because we did not directly measure burnout.

The strengths of our study include a separate focus on the two HA dimensions, an adequate sample size, and the specific focus on HCWs, whose adherence to preventive behaviors is key to protecting themselves and preventing the spreading of COVID-19. However, our study has several limitations. Regardless of the COVID-19 pandemic, washing hands, keeping a safe distance, and wearing a mask are part of everyday safety protocols in every medical environment. While it is possible that HCWs were encouraged to adhere strictly to these routine self-protection behaviors during the pandemic, our findings suggest that a significant proportion of them might have failed to adequately adhere to these preventive behaviors in relation to specific changes in HA. Nevertheless, it is unclear whether what we observed directly resulted from the COVID-19 pandemic, since this was a cross-sectional study with no baseline data for preventive behaviors in the sample during the pre-COVID-19 era; thus, we cannot exclude the possibility that individuals with less adequate preventive behaviors had developed a higher level of HA. We did not identify psychiatric diagnoses, including anxiety and somatic symptom disorders, which could substantially involve HA. Since this study aimed to examine the association of overall compliance with the Japanese government's recommendations for preventive behaviors with HA, the impact of separate preventive behaviors on HA and their relationships to other psychiatric symptoms was not examined and thus may be worth investigating in future studies.

In conclusion, the awfulness dimension of HA, representing the anticipated burden of being ill, was a behavioral marker of poor adherence to preventive behaviors among HCWs during the pandemic. However, the probability dimension, representing the perception of illness likelihood, was not a risk factor for nonadherence to behavioral recommendations. Paying particular attention to the awfulness dimension may help optimize HCWs' self-protection strategies. A two-dimensional understanding of HA may be useful for the maintenance of the healthcare system and public health, as well as HCWs’ own health.

## Data availability statement

The data that support the findings of this study are available from the corresponding authors upon reasonable request.

## Funding

This work was supported by the Japan Health Research Promotion Bureau (JH) Research Fund [grant number 2020-B-09] and the Intramural Research Grant (2-1) for Neurological and Psychiatric Disorders of the 10.13039/501100009438National Center of Neurology and Psychiatry (NCNP).

## Declaration of competing interest

The authors declare the following financial interests/personal relationships which may be considered as potential competing interests:Takeshi Miyama reports financial support was provided by Japan Health Research Promotion Bureau. Kentaro Nagao reports a relationship with 10.13039/501100002975Dainippon Sumitomo Pharma Co Ltd that includes: speaking and lecture fees. Kentaro Nagao reports a relationship with 10.13039/100008373Takeda Pharmaceutical Company Limited that includes: speaking and lecture fees. Takuya Yoshiike reports a relationship with MSD that includes: speaking and lecture fees. Takuya Yoshiike reports a relationship with Takeda Pharmaceutical Company Limited that includes: speaking and lecture fees. Kentaro Matsui reports a relationship with 10.13039/501100003769Eisai Co Ltd that includes: speaking and lecture fees. Kentaro Matsui reports a relationship with 10.13039/501100004169Meiji Seika Pharma Co Ltd that includes: speaking and lecture fees. Kentaro Matsui reports a relationship with MSD that includes: speaking and lecture fees. Kentaro Matsui reports a relationship with 10.13039/501100007132Otsuka Pharmaceutical Co Ltd that includes: speaking and lecture fees. Kentaro Matsui reports a relationship with 10.13039/100008373Takeda Pharmaceutical Company Limited that includes: speaking and lecture fees. Kentaro Matsui reports a relationship with Yoshitomi Pharmaceutical that includes: speaking and lecture fees. Aoi Kawamura reports a relationship with Otsuka Pharmaceutical Co Ltd that includes: speaking and lecture fees. Aoi Kawamura reports a relationship with Takeda Pharmaceutical Company Limited that includes: speaking and lecture fees. Muneto Izuhara reports a relationship with Eisai Co Ltd that includes: speaking and lecture fees. Tomohiro Utsumi reports a relationship with Eisai Co Ltd that includes: speaking and lecture fees. Ayumi Tsuru reports a relationship with MSD that includes: speaking and lecture fees. Hideki Oi reports a relationship with Otsuka Pharmaceutical Co Ltd that includes: funding grants. Hideki Oi reports a relationship with Sumitomo Pharma Co Ltd that includes: funding grants. Hideki Oi reports a relationship with Japan Society of Clinical Trials and Research that includes: board membership. Hideki Oi reports a relationship with Japanese Association of Pharmaceutical Medicine that includes: board membership. Kenichi Kuriyama reports a relationship with Otsuka Pharmaceutical Co Ltd that includes: funding grants. Kenichi Kuriyama reports a relationship with 10.13039/501100005612Shionogi Pharma that includes: funding grants. Kenichi Kuriyama reports a relationship with 10.13039/501100004169Meiji Seika Pharma Co Ltd that includes: funding grants and speaking and lecture fees. Kenichi Kuriyama reports a relationship with Eisai Co Ltd that includes: funding grants and speaking and lecture fees. Kenichi Kuriyama reports a relationship with MSD that includes: funding grants and speaking and lecture fees. Kenichi Kuriyama reports a relationship with Takeda Pharmaceutical Company Limited that includes: funding grants and speaking and lecture fees. Kenichi Kuriyama reports a relationship with 10.13039/501100013420Tsumura & Co that includes: funding grants and speaking and lecture fees. Kenichi Kuriyama reports a relationship with Yoshitomi Pharmaceutical that includes: speaking and lecture fees. Kenichi Kuriyama reports a relationship with Sumitomo Pharma Co Ltd that includes: speaking and lecture fees. Kenichi Kuriyama reports a relationship with Igaku-Shoin Ltd that includes: consulting or advisory. Kazuyuki Nakagome reports a relationship with SHIONOGI that includes: funding grants. Kazuyuki Nakagome reports a relationship with Sumitomo Pharma Co Ltd that includes: funding grants, speaking and lecture fees, and travel reimbursement. Kazuyuki Nakagome reports a relationship with Otsuka Pharmaceutical Co Ltd that includes: funding grants, speaking and lecture fees, and travel reimbursement. Kazuyuki Nakagome reports a relationship with Meiji Seika Pharma Co Ltd that includes: funding grants, speaking and lecture fees, and travel reimbursement. Kazuyuki Nakagome reports a relationship with 10.13039/100008897Janssen Pharmaceuticals Inc that includes: funding grants, speaking and lecture fees, and travel reimbursement. Kazuyuki Nakagome reports a relationship with 10.13039/501100012351Mitsubishi Tanabe Pharma Corporation that includes: funding grants, speaking and lecture fees, and travel reimbursement. Kazuyuki Nakagome reports a relationship with Nippon Boehringer Ingelheim Co Ltd that includes: funding grants, speaking and lecture fees, and travel reimbursement. Kazuyuki Nakagome reports a relationship with Mochida Pharmaceutical Co Ltd that includes: funding grants, speaking and lecture fees, and travel reimbursement. Kazuyuki Nakagome reports a relationship with Takeda Pharmaceutical Company Limited that includes: speaking and lecture fees and travel reimbursement. Kazuyuki Nakagome reports a relationship with 10.13039/501100013327Lundbeck Japan that includes: speaking and lecture fees. Kazuyuki Nakagome reports a relationship with Viatris that includes: speaking and lecture fees. Kazuyuki Nakagome reports a relationship with Eisai Co Ltd that includes: speaking and lecture fees.
