# Supplementary material for: Association between health anxiety dimensions and preventive behaviors during the COVID-19 pandemic among Japanese healthcare workers
Source: Heliyon. 2023 Nov 10;9(11):e22176. doi: 10.1016/j.heliyon.2023.e22176 (PMC10685365; doi:10.1016/j.heliyon.2023.e22176)
Supplement: Multimedia component 1 [file mmc1.docx]

**Table S1. Factors associated with preventive behaviors in participants without physical diseases (n = 416)**

|  | **Crude model** |  | **Adjusted model** |  |
| --- | --- | --- | --- | --- |
| **Variable** | OR (95% CI) | *p* | OR (95% CI) | *p* |
| Age | 1.000 (1.000, 1.001) | 0.313 | 1.000 (0.999, 1.001) | 0.897 |
| Gender |  |  |  |  |
| Men | 1 (ref) |  | 1 (ref) |  |
| Women | 0.993 (0.974, 1.013) | 0.505 | 0.996 (0.976, 1.017) | 0.734 |
| Health anxiety |  |  |  |  |
| Illness likelihood subscale | 1.000 (0.998, 1.002) | 0.660 | 1.002 (0.999, 1.004) | 0.174 |
| Negative consequence subscale | 0.995 (0.990, 1.000) | **0**.**036** | 0.995 (0.990, 1.000) | **0**.**039** |
| Psychological distress | 1.000 (0.998, 1.002) | 0.997 | 1.000 (0.998, 1.002) | 0.943 |
| Sleep disturbance | 0.999 (0.996, 1.003) | 0.722 | 1.000 (0.997, 1.004) | 0.854 |
| Profession |  |  |  |  |
| Director | 1.072 (0.942, 1.221) | 0.293 | 1.088 (0.956, 1.239) | 0.200 |
| Doctor | 1.024 (0.985, 1.065) | 0.238 | 1.022 (0.978, 1.067) | 0.336 |
| Nurse | 0.962 (0.941, 0.983) | **0**.**0004** | 0.965 (0.942, 0.989) | **0**.**004** |
| Other medical staff | 0.981 (0.957, 1.005) | 0.116 | 0.983 (0.959, 1.007) | 0.163 |
| Medical assistant | 0.974 (0.924, 1.026) | 0.322 | 0.985 (0.934, 1.038) | 0.564 |
| Others | 1 (ref) |  | 1 (ref) |  |
| Working position |  |  |  |  |
| Frontline | 0.999 (0.978, 1.020) | 0.934 | 0.996 (0.974, 1.020) | 0.755 |
| Second-line | 1 (ref) |  | 1 (ref) |  |
| History of close contact with COVID-19 cases |  |  |  |  |
| Yes | 1.041 (0.993, 1.092) | 0.096 | 1.068 (1.018, 1.120) | **0**.**007** |
| No | 1 (ref) |  | 1 (ref) |  |
| Body mass index | 1.001 (0.998, 1.003) | 0.712 | 1.000 (0.997, 1.003) | 0.925 |
| Smoking status |  |  |  |  |
| Never | 1 (ref) |  | 1 (ref) |  |
| Former | 1.005 (0.980, 1.031) | 0.699 | 1.008 (0.982, 1.034) | 0.560 |
| Current | 0.980 (0.949, 1.011) | 0.195 | 0.981 (0.949, 1.014) | 0.247 |
| Alcohol intake |  |  |  |  |
| Non-drinkers (< 1 day/month) | 1 (ref) |  | 1 (ref) |  |
| Occasional drinkers (1–3 days/month) | 1.010 (0.984, 1.038) | 0.444 | 1.010 (0.984, 1.037) | 0.442 |
| Regular drinkers (1–2 days/week or more) | 1.010 (0.990, 1.031) | 0.312 | 1.009 (0.988, 1.029) | 0.407 |
| Exercise habits |  |  |  |  |
| < 1 hour per week | 1 (ref) |  | 1 (ref) |  |
| ≥ 1 hour per week | 1.027 (1.007, 1.046) | **0**.**006** | 1.020 (1.001, 1.040) | **0**.**043** |
| OR, odds ratio; CI, confidence interval; COVID-19, coronavirus disease 2019 | | | | |

**Table S2. Factors associated with preventive behaviors in all participants after multiple imputation (N = 657)**

|  | **Crude model** |  | **Adjusted model** |  |
| --- | --- | --- | --- | --- |
| **Variable** | OR (95% CI) | *p* | OR (95% CI) | *p* |
| Age | 1.000 (1.000, 1.001) | 0.165 | 1.000 (0.999, 1.001) | 0.851 |
| Gender |  |  |  |  |
| Men | 1 (ref) |  | 1 (ref) |  |
| Women | 0.996 (0.981, 1.012) | 0.643 | 0.994 (0.977, 1.011) | 0.483 |
| Health anxiety |  |  |  |  |
| Illness likelihood subscale | 1.000 (0.999, 1.002) | 0.586 | 1.001 (1.000, 1.003) | 0.080 |
| Negative consequence subscale | 0.994 (0.990, 0.997) | **0**.**001** | 0.993 (0.989, 0.997) | **0**.**0005** |
| Psychological distress | 1.000 (0.998, 1.001) | 0.816 | 1.000 (0.999, 1.002) | 0.652 |
| Sleep disturbance | 0.999 (0.997, 1.002) | 0.619 | 0.999 (0.996, 1.002) | 0.580 |
| Profession |  |  |  |  |
| Director | 1.010 (0.936, 1.190) | 0.791 | 1.010 (0.936, 1.089) | 0.803 |
| Doctor | 1.010 (0.979, 1.042) | 0.537 | 0.999 (0.966, 1.033) | 0.945 |
| Nurse | 0.975 (0.958, 0.992) | **0**.**003** | 0.971 (0.953, 0.990) | **0**.**003** |
| Other medical staff | 0.975 (0.956, 0.995) | **0**.**013** | 0.976 (0.957, 0.997) | **0**.**022** |
| Medical assistant | 0.957 (0.915, 1.001) | 0.054 | 0.966 (0.924, 1.009) | 0.117 |
| Others | 1 (ref) |  | 1 (ref) |  |
| Working position |  |  |  |  |
| Frontline | 1.010 (0.994, 1.027) | 0.224 | 1.012 (0.994, 1.031) | 0.183 |
| Second-line | 1 (ref) |  | 1 (ref) |  |
| History of close contact with COVID-19 cases |  |  |  |  |
| Yes | 1.043 (1.004, 1.084) | **0**.**029** | 1.063 (1.022, 1.105) | **0**.**002** |
| No | 1 (ref) |  | 1 (ref) |  |
| Body mass index | 1.001 (0.999, 1.003) | 0.322 | 1.000 (0.998, 1.002) | 0.789 |
| Smoking status |  |  |  |  |
| Never | 1 (ref) |  | 1 (ref) |  |
| Former | 1.011 (0.991, 1.030) | 0.188 | 1.010 (0.990, 1.030) | 0.324 |
| Current | 0.980 (0.955, 1.005) | 0.075 | 0.980 (0.954, 1.007) | 0.141 |
| Alcohol intake |  |  |  |  |
| Non-drinkers (< 1 day/month) | 1 (ref) |  | 1 (ref) |  |
| Occasional drinkers (1–3 days/month) | 1.000 (0.980, 1.021) | 0.968 | 0.996 (0.976, 1.016) | 0.680 |
| Regular drinkers (1–2 days/week or more) | 1.000 (0.984, 1.017) | 0.983 | 0.998 (0.982, 1.014) | 0.775 |
| Current physical diseases |  |  |  |  |
| Yes | 0.011 (0.995, 1.028) | 0.185 | 1.004 (0.989, 1.020) | 0.572 |
| No | 1 (ref) |  | 1 (ref) |  |
| Exercise habits |  |  |  |  |
| < 1 hour per week | 1 (ref) |  | 1 (ref) |  |
| ≥ 1 hour per week | 1.010 (0.995, 1.026) | 0.182 | 1.008 (0.991, 1.025) | 0.376 |
| OR, odds ratio; CI, confidence interval; COVID-19, coronavirus disease 2019 | | | | |
