# Supplementary material for: Association between health anxiety dimensions and preventive behaviors during the COVID-19 pandemic among Japanese healthcare workers
Source: Heliyon. 2023 Nov 10;9(11):e22176. doi: 10.1016/j.heliyon.2023.e22176 (PMC10685365; doi:10.1016/j.heliyon.2023.e22176)
Supplement: Multimedia component 2 [file mmc2.docx]

**Appendix I. Survey questionnaire for the current study (English version)**

Q. Sex:

- Male
- Female

Q. Age:

- ＿＿years old

Q. Height:

- ＿＿centimeters

Q. Body weight:

- ＿＿kilograms

Q. What is your profession?

- Director
- Physician, Dentist
- Nurse, Assistant Nurse, Midwife, Public Health Nurse
- Other medical staff
- Medical assistant
- General office worker
- Information manager
- Worker in the sector of research, development, international cooperation, education
- Facility service worker (cleaning, laundry, garbage disposal, security, etc.)
- Personal service worker other than medical services (food and beverage, sales, barbering/beauty, cleaning, etc.)
- Others (Please specify: _________________?

Q. Have you been involved in the treatment of patients with COVID-19?

- Yes
- No

Q. Are you vaccinated against COVID-19?

- Yes
- No

Q. Have you ever had close contact with a patient with COVID-19? Close contact is defined as living with someone who had COVID-19; direct physical contact with a person with COVID-19; providing direct care for patients with COVID-19 or being in direct contact with the patient’s secretions without using proper personal protective equipment; or spending more than 15 minutes within one meter of a person with COVID-19.

- Yes
- No

Q. In the last month, how often have you performed each of the following preventive behaviors?

- Always
- Usually
- Seldom
- Never

1. Avoiding three places/situations (Closed spaces with poor ventilation, Crowded places with many people nearby, and Close-contact settings such as close-range conversations)
2. Staying at least two meters (1 meter if it is difficult) away from others
3. Wearing a mask when talking outside or indoors
4. Adhering to hand hygiene (hand washing and hand disinfection) when returning from outside or before eating

Q. Smoking:

- Never
- Former
- Sometimes
- every day (less than 10 cigarettes a day)
- every day (11-20 cigarettes a day)
- every day (21 or more cigarettes a day)

Q. How often do you drink alcohol (e.g., sake, shochu, beer, Western liquor)?

- < 1 day a month
- 1-3 days a month
- 1-2 days a week
- 3-4 days a week
- 5-6 days a week

Q. How much exercise (including walking and stretching) do you do on weekends or when you have time?

- Never
- Less than 30 minutes per week
- 30-59 minutes per week
- More than 1 hour but less than 2 hours per week
- More than 2 hours but less than 3 hours per week
- More than 3 hours but less than 4 hours per week
- More than 4 hours per week

Q. Do you have any of the following medical conditions?

- No
- Yes and under treatment
- Yes but not under treatment

1. Hypertension
2. Diabetes
3. Chronic lung diseases (e.g., chronic obstructive pulmonary disease, bronchial asthma)
4. Cardiac disease
5. Cerebrovascular disease
6. Cancer
7. Other chronic diseases (please specify___________)

Q. During the last 30 days, about how often did you feel …

|  | All of the time | Most of the time | Some of the time | A little of the time | None of the time |
| --- | --- | --- | --- | --- | --- |
| a. …nervous? | 1 | 2 | 3 | 4 | 5 |
| b. …hopeless? | 1 | 2 | 3 | 4 | 5 |
| c. …restless or fidgety? | 1 | 2 | 3 | 4 | 5 |
| d. …so depressed that nothing could cheer you up? | 1 | 2 | 3 | 4 | 5 |
| e. …that everything was an effort? | 1 | 2 | 3 | 4 | 5 |
| f. …worthless? | 1 | 2 | 3 | 4 | 5 |

Q. Each question consists of a group of four statements. Please read each group of statements carefully and then select the one which best describes your feelings, over the past six months. Identify the statement by ringing the letter next to it, i.e. if you think that statement (a) is correct, ring statement (a); it may be that more than one statement applies, in which case, please ring any that are applicable.

|  | |
| --- | --- |
| 1 | (a) I do not worry about my health.  (b) I occasionally worry about my health.  (c) I spend much of my time worrying about my health.  (d) I spend most of my time worrying about my health. |
| 2 | (a) I notice aches}pains less than most other people (of my age).  (b) I notice aches}pains as much as most other people (of my age).  (c) I notice aches}pains more than most other people (of my age).  (d) I am aware of aches}pains in my body all the time. |
| 3 | (a) As a rule I am not aware of bodily sensations or changes.  (b) Sometimes I am aware of bodily sensations or changes.  (c) I am often aware of bodily sensations or changes.  (d) I am constantly aware of bodily sensations or changes. |
| 4 | (a) Resisting thoughts of illness is never a problem.  (b) Most of the time I can resist thoughts of illness.  (c) I try to resist thoughts of illness but am often unable to do so.  (d) Thoughts of illness are so strong that I no longer even try to resist them. |
| 5 | (a) As a rule I am not afraid that I have a serious illness.  (b) I am sometimes afraid that I have a serious illness.  (c) I am often afraid that I have a serious illness.  (d) I am always afraid that I have a serious illness. |
| 6 | (a) I do not have images (mental pictures) of myself being ill.  (b) I occasionally have images of myself being ill.  (c) I frequently have images of myself being ill.  (d) I constantly have images of myself being ill. |
| 7 | (a) I do not have any difficulty taking my mind off thoughts about my health.  (b) I sometimes have difficulty taking my mind off thoughts about my health.  (c) I often have difficulty in taking my mind off thoughts about my health.  (d) Nothing can take my mind off thoughts about my health. |
| 8 | (a) I am lastingly relieved if my doctor tells me there is nothing wrong.  (b) I am initially relieved but the worries sometimes return later.  (c) I am initially relieved but the worries always return later.  (d) I am not relieved if my doctor tells me there is nothing wrong. |
| 9 | (a) If I hear about an illness I never think I have it myself.  (b) If I hear about an illness I sometimes think I have it myself.  (c) If I hear about an illness I often think I have it myself.  (d) If I hear about an illness I always think I have it myself. |
| 10 | (a) If I have a bodily sensation or change I rarely wonder what it means.  (b) If I have a bodily sensation or change I often wonder what it means.  (c) If I have a bodily sensation or change I always wonder what it means.  (d) If I have a bodily sensation or change I must know what it means. |
| 11 | (a) I usually feel at very low risk for developing a serious illness.  (b) I usually feel at fairly low risk for developing a serious illness.  (c) I usually feel at moderate risk for developing a serious illness.  (d) I usually feel at high risk for developing a serious illness. |
| 12 | (a) I never think I have a serious illness.  (b) I sometimes think I have a serious illness.  (c) I often think I have a serious illness.  (d) I usually think that I am seriously ill. |
| 13 | (a) If I notice an unexplained bodily sensation I don’t find it difficult to think about other things.  (b) If I notice an unexplained bodily sensation I sometimes find it difficult to think about other things.  (c) If I notice an unexplained bodily sensation I often find it difficult to think about other things.  (d) If I notice an unexplained bodily sensation I always find it difficult to think about other things. |
| 14 | (a) My family/friends would say I do not worry enough about my health.  (b) My family/friends would say I have a normal attitude to my health.  (c) My family/friends would say I worry too much about my health.  (d) My family/friends would say I am a hypochondriac. |
| For the following questions, please think about what it might be like if you had a serious illness of a type which particularly concerns you (such as heart disease, cancer, multiple sclerosis and so on). Obviously you cannot know for definite what it would be like; please give your best estimate of what you think might happen, basing your estimate on what you know about yourself and serious illness in general. | |
| 15 | (a) If I had a serious illness I would still be able to enjoy things in my life quite a lot.  (b) If I had a serious illness I would still be able to enjoy things in my life a little.  (c) If I had a serious illness I would be almost completely unable to enjoy things in my life.  (d) If I had a serious illness I would be completely unable to enjoy life at all. |
| 16 | (a) If I developed a serious illness there is a good chance that modern medicine would be able to cure me.  (b) If I developed a serious illness there is a moderate chance that modern medicine would be able to cure me.  (c) If I developed a serious illness there is a very small chance that modern medicine would be able to cure me.  (d) If I developed a serious illness there is no chance that modern medicine would be able to cure me. |
| 17 | (a) A serious illness would ruin some aspects of my life.  (b) A serious illness would ruin many aspects of my life.  (c) A serious illness would ruin almost every aspect of my life.  (d) A serious illness would ruin every aspect of my life. |
| 18 | (a) If I had a serious illness I would not feel that I had lost my dignity.  (b) If I had a serious illness I would feel that I had lost a little of my dignity.  (c) If I had a serious illness I would feel that I had lost quite a lot of my dignity.  (d) If I had a serious illness I would feel that I had totally lost my dignity. |

The following questions relate to your usual sleep habits during the past month only. Your answers should indicate the most accurate reply for the majority of days and nights in the past month. Please answer all questions.

1. During the past month, what time have you usually gone to bed at night?

- AM/PM __:__

1. During the past month, how long (in minutes) has it usually taken you to fall asleep each night?

- About ____ minutes

1. During the past month, what time have you usually gotten up in the morning?

- AM/PM __:__

Q. During the past month, how many hours of actual sleep did you get at night? (This may be different than the number of hours you spent in bed.)

- __:__

| Q.During the past month, how often have you had trouble sleeping because you... | Not during the past month | Less than once a week | Once or twice a week | Three or more times a week |
| --- | --- | --- | --- | --- |
| A. Cannot get to sleep within 30 minutes | 0 | 1 | 2 | 3 |
| B. Wake up in the middle of the night or early morning | 0 | 1 | 2 | 3 |
| C. Have to get up to use the bathroom | 0 | 1 | 2 | 3 |
| D. Cannot breathe comfortably | 0 | 1 | 2 | 3 |
| E. Cough or snore loudly | 0 | 1 | 2 | 3 |
| F. Feel too cold | 0 | 1 | 2 | 3 |
| G. Feel too hot | 0 | 1 | 2 | 3 |
| H. Have bad dreams | 0 | 1 | 2 | 3 |
| I. Have pain | 0 | 1 | 2 | 3 |
| J. Other reason(s), please describe:_______ | 0 | 1 | 2 | 3 |

Q. During the past month, how would you rate your sleep quality overall?

- Very good
- Fairly good
- Fairly bad
- Very bad

Q. During the past month, how often have you taken medicine to help you sleep (prescribed or “over the counter”)?

- Not during the past month
- Less than once a week
- Once or twice a week
- Three or more times a week

Q. During the past month, how often have you had trouble staying awake while driving, eating meals, or engaging in social activity?

- Not during the past month
- Less than once a week
- Once or twice a week
- Three or more times a week

Q. During the past month, how much of a problem has it been for you to keep up enough enthusiasm to get things done?

- No problem at all
- Only a very slight problem
- Somewhat of a problem
- A very big problem
